# A Complete Molecular Diagnostic Procedure for Applications in Surveillance and Subtyping of Avian Influenza Virus

**DOI:** 10.1155/2014/653056

**Published:** 2014-06-26

**Authors:** Chun-Hsien Tseng, Hsiang-Jung Tsai, Chung-Ming Chang

**Affiliations:** ^1^Animal Health Research Institute, Danshui, Taipei 25158, Taiwan; ^2^Graduate Institute of Veterinary Medicine, College of Bioresource and Agriculture, National Taiwan University, No. 1, Section 4, Roosevelt Road, Taipei 106, Taiwan; ^3^Research Center for Emerging Viral Infections, Department of Medical Biotechnology and Laboratory Science, College of Medicine, Chang Gung University, Kwei-Shan, Taoyuan 333, Taiwan

## Abstract

*Introduction*. The following complete molecular diagnostic procedure we developed, based on real-time quantitative PCR and traditional PCR, is effective for avian influenza surveillance, virus subtyping, and viral genome sequencing. *Method*. This study provides a specific and sensitive step-by-step procedure for efficient avian influenza identification of 16 hemagglutinin and 9 neuraminidase avian influenza subtypes. *Result and Conclusion*. This diagnostic procedure may prove exceedingly useful for virological and ecological advancements in global avian influenza research.

## 1. Introduction

In recent years, the appearance of highly pathogenic avian influenza viruses (AIVs) among poultry, mammalian, and human populations has raised serious public health concerns worldwide. With reports of H5N1 emerging from North Africa and Central and South Asia, the fear of continued recombination introducing a pandemic strain of AIV has prompted many organizations to place a greater emphasis on the development of surveillance and epidemiological techniques [1, 2]. Efficient identification allows for a better understanding of behavior, movement, and interaction of AIV reservoir species (e.g., waterfowl such as* anseriformes* and* charadriiformes*) and development of more successful containment and quarantine strategies for more timely medical intervention [3–5].

As members of the* Orthomyxoviridae *family, Influenza A viruses are made up of eight gene segments and classified according to the combination of hemagglutinin (HA) and neuraminidase (NA) proteins found on their membrane surface [6, 7]. Currently, 16 subtypes of HA and 9 subtypes of NA have been identified from both wild and domestic avians [8]. A distinct H17 subtype lineage has also been isolated from bats [9]. Given the importance of these surface proteins in cell entry and exit, identification of the viral subtype can often provide valuable insight into the origin, present course, and infectivity of the virus [6].

To properly identify the HA and NA subtypes, many diagnostic laboratories have replaced traditional methods (i.e., replication in embryonated eggs or tissues followed by HA/NA inhibition assays) with molecular approaches such as reverse transcription polymerase chain reaction (RT-PCR). This procedure provides a specific, sensitive, and quick method of characterizing AIV subtypes while maintaining easy and cost-effective means [10–12]. Present discussions of molecular methods have a tendency to identify HA and NA separately [6, 13–15]. Therefore this study describes a more expansive molecular diagnostic procedure for avian influenza viral subtyping and sequencing that may help researchers to perform comprehensive approaches on influenza surveillance and virus subtype identification.

## 2. Material and Methods

### 2.1. Avian Influenza Reference Viruses

Seventeen avian influenza viruses that contain 16 HA and 9 NA subtypes were selected to serve as reference strains (Table 1).

### 2.2. Influenza Virus Confirmation by a One-Step Taqman Real-Time Quantitative RT-PCR

The presence of influenza virus was confirmed by subjecting each reference sample to a one-step real-time Taqman quantitative RT-PCR (Taqman RT-qPCR), targeting the M gene for AIV detection. The one-step real-time Taqman RT-qPCR was performed with the Roche LightCycler 480 system using the LightCycler 480 Probe Master kit (Roche). Reaction was carried out in a final volume of 10 *μ*L containing 0.2 *μ*M of Taqman probe: 5′-(6-Fam) CCT CAA AGC CGA GAT CGC GCA (Tamra)-3′, 0.5 *μ*M of forward primer: FluA-M52C (5′-CTT CTA ACC GAG GTC GAA ACG-3′), 0.5 *μ*M of reverse primer: FluA-M253R (5′-AGG GCA TTT TGG ACA AAK CGT CTA-3′) [16], 1x LighterCycler 480 Probes Master, and 1 *μ*g of template viral RNA. Conditions of amplification were as follows: reverse transcription at 50°C for 2 min, denaturation at 95°C for 2 min, followed with 40 cycles of amplification (10 seconds at 95°C and 30 seconds at 60°C), and cooling at 40°C.

### 2.3. Primer Design for Virus Subtyping

More than 2000 HA and NA sequences were retrieved from the GenBank of the National Center of Biotechnology Information (NCBI) in order to identify conserved regions for primer design.* Sequencher* (Gene Codes Corporation) and* BioEdit* (Tom Hall) were used to align and analyze these sequences. A total of 25 specific primer pairs were created to specifically target each of the 16 HA and 9 NA subtypes (see Supplemental Table 1 in Supplementary Material available online at http://dx.doi.org/10.1155/2014/653056).

### 2.4. Viral RNA Extraction

Viral RNA of each reference strain was extracted using the Viral RNA Mini Kit (Qiagen) according to the manufacturer's instructions. Viral RNA extraction was performed from 120 *μ*L of specimen and the viral RNA was eluted into a final volume of 60 *μ*L RNase-free water. Viral RNA extracted from reference strains or clinical specimens served as template viral RNA for one-step Taqman real-time RT-qPCR and also for reverse transcription to produce cDNA for PCR reactions. Extracted viral RNA not used immediately was stored at −80°C.

### 2.5. Reverse Transcription (RT)

Each reverse transcription reaction had a final volume of 20 *μ*L containing 5 units of ReverTra Ace RTase (TOYOBO), 0.5 *μ*M of Uni12 primer: 5′-AGCAAAAGCAGG-3′, 1 mM of dNTP mixture, 20 units of RNase inhibitor (Promega), and 1 *μ*g of template viral RNA. The reaction ran for 45 min at 37°C, followed by 15 min at 42°C and 15 min at 95°C.

### 2.6. Two-Step SYBR-Green Real-Time PCR for Virus Subtyping

Real-time PCR was carried out in Roche LightCycler 480 system using a LightCycler 480 SYBR-Green I Master kit (Roche) in a final volume of 10 *μ*L mixture containing 1x PCR buffer, 0.5 *μ*M of forward and reverse primers, and 1 *μ*L of template cDNA from RT reaction. The PCR conditions ran at 95°C for 5 min, followed with 40 cycles of amplification at 95°C for 10 sec, 60°C for 10 sec, and 72°C for 20 sec, with a final extension at 72°C for 1 min. Melting curve analyses were performed on the PCR products by progressive heating from 60°C to 95°C. Differences in melting curves were used to distinguish virus subtypes.

### 2.7. Standard Curve of Detection System

The concentration of H4N8 reference strain viral RNA was determined by NanoDrop (Thermo). Standard curves of Taqman RT-qPCR for avian influenza M gene were established on 10-fold serial dilutions of influenza viral RNA with duplicate testing from 0.1 ng/mL to 1 fg/mL. Standard curves of SYBR-Green qPCR for M, HA, and NA genes were established on 10-fold serial dilutions of reverse transcribed cDNA of reference viral RNA from 5 pg/mL to 1 ag/mL to estimate in duplicate the system's liner range, efficiency, and detection limitation.

### 2.8. Traditional PCR and Direct Sequencing for Virus Sequence Analysis

**Figure 1:**
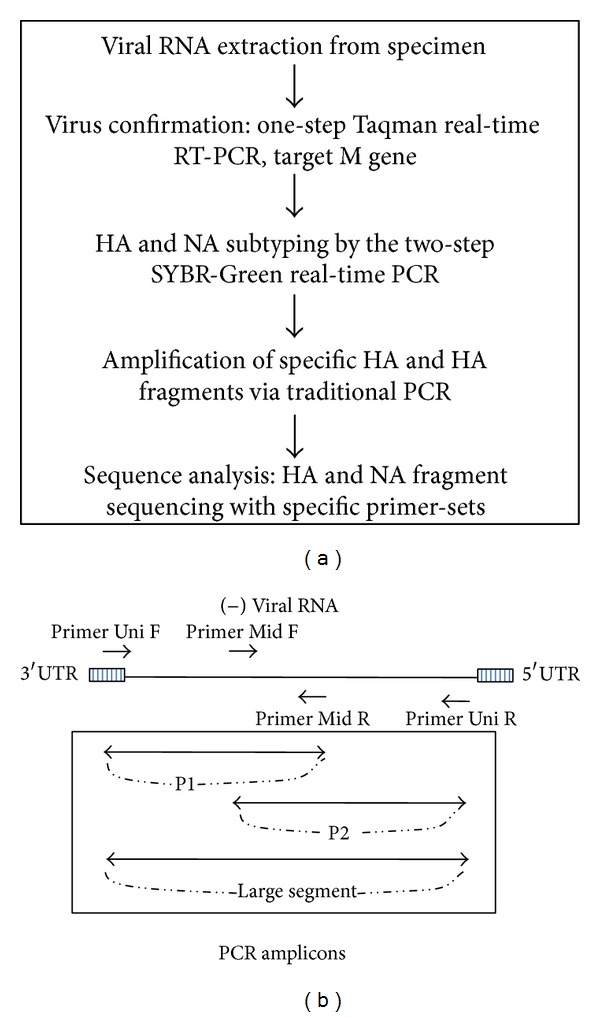
(a) Flowchart outlining the molecular diagnostic procedure: from viral RNA extraction to HA and NA fragments sequencing and analysis. (b) Diagram showing the related positions of specific primers and the three PCR amplicons (P1, P2, and F) during the traditional PCR amplification step. UTR: untranslated region. P1: partial 1 fragment (primers Uni12 F and Mid R). P2: partial 2 fragment (primers Mid F and Uni12 R). F: large fragment (primers Uni12 F and Uni12 R).

An additional 100 primers were selected for sequencing of all 16 HA and 9 NA subtypes (Supplemental Table 2). To obtain the complete HA and NA gene sequences, primer-sets enclosed either the entire fragment (full length, F) or the two overlapping regions (P1 and P2) (Figure 1(b)). Each PCR reaction was carried out in a final volume of 25 *μ*L containing 1x PCR buffer, 2.5 units of Taq DNA polymerase recombinant (Invitrogen), 0.2 *μ*M of dNTP, 1.5 mM of MgCl_2_, 0.5 *μ*M of each primer-set, and 5 *μ*L of template cDNA.

PCR conditions for most HA gene segments consisted of 3 minutes at 94°C, followed by 40 cycles of 30 sec at 94°C, 30 sec at 54°C, 3 min at 72°C, and a final extension of 7 min at 72°C. Two groups of HA amplicons (H1-P1, H4-F, H5-P2, H14-P1 and H3-P1, H6-P2, H9-P1, H13-P2, H14-P2, H16-P2) had a modified annealing temperature of 57°C and 50°C, respectively. PCR conditions for NA gene segments were set to 3 min at 94°C, 30 sec at 54°C, 2 min at 72°C, and a final extension of 7 minutes at 72°C.

PCR products were separated by gel electrophoresis using a 1.5% agarose gel made with 0.5x TBE. Reference ladder and PCR products were prestained with SYBR-Green. DNA fragments of interest were purified using the gel extraction kit (Qiagen) and sent for direct sequencing. Subsequent sequence analysis and alignments were performed using* BioEdit *(Tom Hall) and compared against database sequences in the GenBank.

## 3. Results

### 3.1. Establishment of a Complete Avian Influenza Virus Diagnostic Procedure

This study outlines a comprehensive step-by-step system for the viral subtype and sequence identification of all HA and NA avian influenza subtypes (Figure 1(a)). First, the presence of AIV was observed by a one-step Taqman RT-qPCR to detect the conserved M gene segment. The subtype of the influenza was then determined by running 25 separate SYBR-Green real-time PCR reactions (16 HA and 9 NA), followed by melting curve analyses. Based on these results, specific primer-sets (Supplemental Table 2) are chosen and used to amplify the HA and NA genes via traditional PCR and sent for direct sequencing to obtain data needed for further study and classification.

### 3.2. Two-Step SYBR-Green Real-Time qPCR

**Figure 2:**
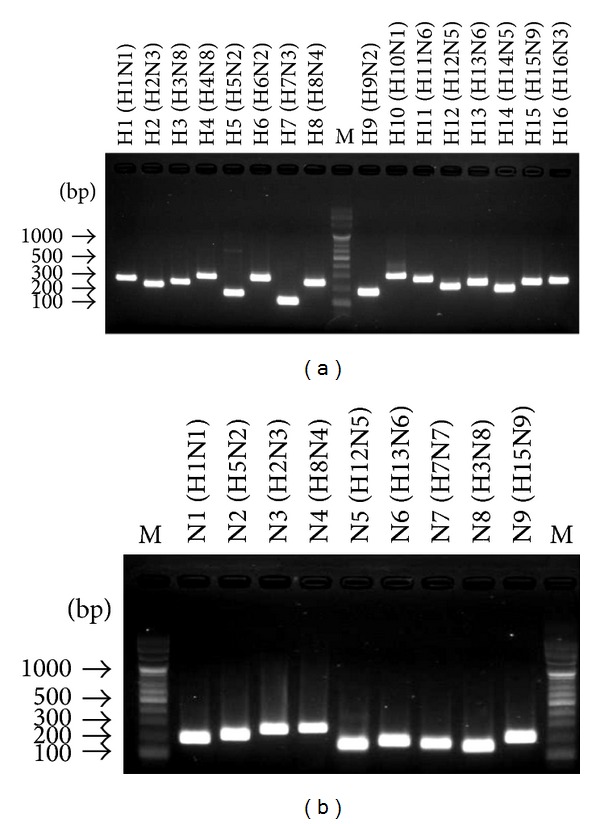
Gel electrophoresis; visual confirmation of SYBR-Green real-time qPCR subtyping results. (a) 16 HA single PCR amplicons for each subtype with high efficiency. (b) 9 NA single PCR amplicons for each subtype with high efficiency. M represents the 100 bps DNA marker.

At least three primer-sets for each HA and NA were designed and tested for SYBR-Green real-time qPCR in this study. Those HA and NA primer-sets which produced nonspecific amplifications or showed cross-reactions with other nontarget virus strains were eliminated. Each selected HA (H1-H16) and NA (N1-N9) subtyping primer-set was tested in heterologous combination conditions with all reference virus strains (e.g., H1N1 virus tested by all H1-H16 primer-sets) to determine its specificity. The HA and NA primer-set which generated only one specific single melting wave in SYBR-Green real-time qPCR was decided to be the specific subtyping primer-set. The specificity of the subtyping primer panel was highly efficient as only a single SYBR-Green melting wave was generated for each HA and NA subtype. Successful products were confirmed through gel electrophoresis (Figure 2). Subtyping results were double-checked by comparing the sequenced PCR amplicons against reference sequences supplied by GenBank.

### 3.3. Sequence Analysis of HA and NA PCR Fragments

**Figure 3:**
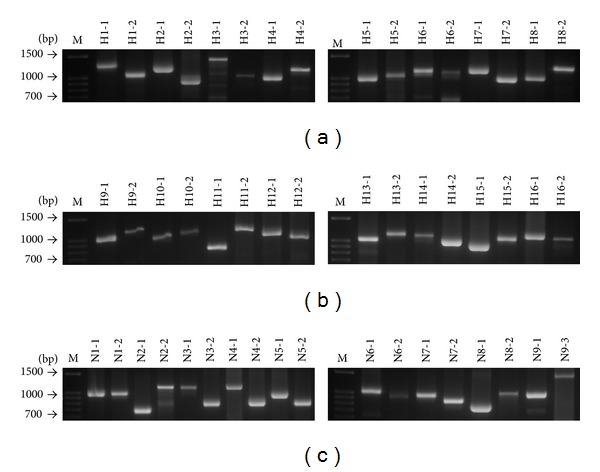
Gel electrophoresis; full length or overlapping partial fragments of 16 HA and 9 NA traditional PCR amplicons for sequencing analysis.

Full length, or nearly full length, of HA and NA sequence data was successfully obtained by analyzing either the full length fragment, F, or the overlapping partial fragments, P1 and P2 (Figure 3). Together, these PCR products allowed for the identification and sequencing of approximately 1,700 bases of the HA gene and 1,400 bases of the NA gene. Comparison with reference database sequences suggests that amplification of HA and/or NA coding regions was highly efficient (Table 1).

### 3.4. Standard Curve of Detection System

**Figure 4:**
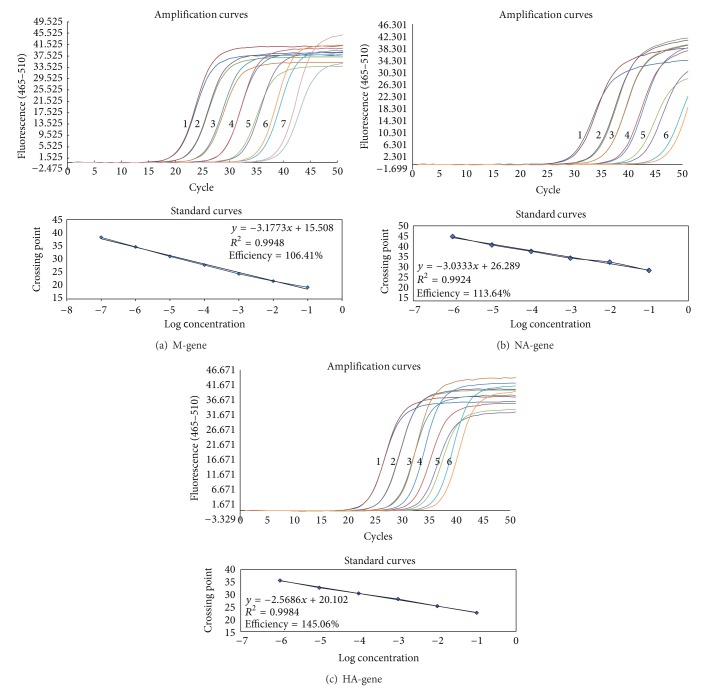
Amplification and standard curves of two-step SYBR-Green qPCR for influenza virus detection and subtyping using reference H4N8 stain as model. (a) M gene, (b) NA gene, (c) HA gene; (i) cDNA template concentration in each amplification curve: (1) 5 pg/mL; (2) 0.5 pg/mL; (3) 0.05 pg/mL; (4) 5 fg/mL; (5) 0.5 fg/mL; (6) 0.05 fg/mL; (7) 5 ag/mL. (ii) Correlation coefficient and PCR efficiency for each gene were detailed in each standard curves' chart.

One-step Taqman qRT-PCR for avian influenza M gene detection achieved liner range from 0.1 ng/mL to 0.01 pg/mL; PCR efficiency was 99.75 percent and the detection limit was 0.01 pg/mL (data not shown). Two-step SYBR-Green qPCR for avian influenza M gene detection achieved liner range from 5 pg/mL to 5 ag/mL; qPCR efficiency was 106.4 percent and the detection limit was 5 ag/mL. SYBR-Green qPCR for both avian influenza HA and NA gene detection achieved liner range from 5 pg/mL to 0.05 fg/mL; qPCR efficiency is 113.64 percent for NA gene detection, and 145.06 percent for HA gene detection. The SYBR-Green detection limit for HA and HA gene is 0.05 fg/mL (Figure 4).

## 4. Discussion

Since the development of molecular biological techniques, such as multiplex reverse transcription PCR [17, 18], real-time reverse transcription PCR [19], and high-resolution melting curve analysis [20], the speed and efficiency of avian influenza virus detection have improved drastically in recent years. However, due to continuous antigenic shift and drift, these procedures remain limited by the constant need to redesign primers/probes in response to the high evolution rate of influenza viruses. Without revision, the use of outdated primers might result in being unable to identify rare or emerging viral strains [17].

Fortunately, problems mentioned above can be solved in our diagnostic procedure because we combine the advantages of Taqman RT-qPCR, SYBR-Green qPCR, traditional PCR, and sequencing analysis. The advantages of SYBR-Green qPCR are that it is less expensive compared to TaqMan RT-qPCR, easy to use, and sensitive. Using SYBR-Green qPCR for AI subtype screening at the first step of diagnostic procedure can greatly reduce the disadvantage of primers/probes redesign problem because there is no probe needed in SYBR-Green qPCR system. Small fragment is easier to amplify than long fragment in PCR reactions. In clinical conditions, the virus concentration from swabs specimen can be just high enough for Taqman RT-qPCR for avian influenza detection on M gene but is too low for traditional PCR reactions to work well to amplify long fragments for HA or NA sequence analysis. The M gene PCR amplicon is 202 base pairs. When the original virus material from swab samples is limited and the traditional PCR does not work well to amplify directly the nearly full length of HA and NA sequences, 1,700 and 1,400 base pairs, respectively, or HA and NA overlapping partial P1 and P2 fragments, around 800–1,100 base pairs (Figure 3), the alternative nest-PCR approach can be performed to solve this problem. The alternative nest-PCR is performed by using the first primer-sets to amplify the nearly full length of HA or NA sequence in the first phase of amplification and then the partial P1 and P2 primer-sets are used to prime within the first PCR product to produce high concentration of shorter amplicons of partial HA and NA sequences. Definitely, primer-sets of SYBR-Green qPCR for AI subtype determination need to be designed in the conserved region of virus sequence for each HA and NA subtype.

From the standard curve results, it looks like that the two-step SYBR-Green qPCR system for avian M gene detection is more sensitive than the one-step Taqman RT-qPCR system, because the detection limit is 5 ag/mL for SYBR-Green system and 0.01 pg/mL for Taqman system. In fact, it is not appropriate to compare the sensitivity of two different systems together because these two systems were designed for different purposes. The one-step Taqman RT-qPCR in this study was designed for detection of avian influenza virus because of its specificity and its rapidity. The one-step Taqman RT-qPCR system for avian influenza detection with a specific probe for M gene can greatly increase the detection specificity and reduce the detection time because no reverse transcription procedure is needed compared to SYBR-Green system. The efficiency of SYBR-Green qPCR system for HA and NA is higher than 100% that can be explained by nonspecific products, like primer-dimer, being amplified and detected by SYBR-Green system. But this phenomenon does not affect the specificity of subtyping procedure.

It must be noted that although the use of SYBR-Green qPCR runs the risk of cross-reaction, it did not hinder the isolation and identification of HA and NA subtypes. Band-size comparisons and database analyses can serve as a simple validation, thereby providing a confident means of differentiating between side and target products. Our procedure proposes that in place of a single amplicon, two or three PCR products (F, P1, and P2) be targeted for amplification (Figure 1(b)). Given the large region of overlap shared between the three target amplicons, the procedure provided a comprehensive method to break through the limitation with the use of worldwide reference strains of HA and NA. Furthermore, this procedure permits a greater conservation of resources using a final volume of 10 *μ*L compared to the usual 20–25 *μ*L. This study has effectively demonstrated complete diagnostic procedure based on two-step SYBR-Green real-time RT-PCR and melting curve analysis can correctly differentiate all 16 HA and 9 NA avian influenza virus subtypes.

This procedure has been performed and proved very useful for avian influenza surveillance of wild birds, both in migratory birds and in resident birds, form cloacal and tracheal samples. In the recent study, cloacal and tracheal samples have been tested by this procedure, and different subtypes of low-pathogenic avian influenza viruses have been identified from common teal (*Anas crecca*), cattle egret (*Bubulcus ibis*), long-toed stint (*Calidris subminuta*), kentish plover (*Charadrius alexandrinus*), and little egret (*Egretta garzetta*) and the avian influenza positive rate was 2.17%. (unpublished data). Through collaboration with effective AIV capture methods [21], this procedure can have additional applications in easing the collection and analysis of data for environmental/epidemiological surveys. Results obtained more complete (updates current) HA and NA sequences providing further uses in the identification of antiviral drug resistant strains, as well as in the tracking of viral evolution over time.

## Supplementary Material

Supplemental Table 1 details 25 SYBR-Green qPCR detection primer pairs targeting on 16 HA and 9 NA subtypes of avian influenza. Supplemental Tale 2 lists the detail of 100 primers which are designed for traditional PCR and sequencing purposes for different subtypes of avian influenza virus sequence analysis.

## Figures and Tables

**Table 1:** Reference strains used in this study.

Reference strains	Accession number of HA gene	Accession number of NA gene
A/Duck/Italy/1447/2005 (H1N1)	GQ247841	GQ247842
A/Duck/Germany/1215/1973 (H2N3)	GQ247843	GQ247844
A/psittacine/Italy/2873/2000 (H3N8)	GQ247846	GQ247845
A/Cockatoo/England/1972 (H4N8)	GQ247847	GQ247848
A/Turkey/Italy/1980 (H5N2)	GQ247849	GQ247850
A/Turkey/Canada/63 (H6N2)	GQ247851	GQ247852
A/Turkey/Italy/9289/V02 (H7N3)	GQ247853	GQ247854
A/Macaw/Italy/626/80 (H7N7)	Non determined	GQ247855
A/Turkey/Ontario/6118/1968 (H8N4)	GQ247856	GQ247857
A/Turkey/Wisconsin/1/1966 (H9N2)	GQ247858	GQ247859
A/Ostrich/SA/2001 (H10N1)	GQ247860	GQ247861
A/Duck/England/1/1956 (H11N6)	GQ247862	GQ247863
A/Duck/Alberta/60/1976 (H12N5)	GQ247864	GQ247865
A/Gull/Maryland/704/1977 (H13N6)	GQ247866	GQ247867
A/Mallard/Astrakhan/263/1982 (H14N5)	GQ247868	GQ247869
A/Shearwater/West Australia/2576/79 (H15N9)	GQ247870	GQ247871
A/Gull/Denmark/68110/2002 (H16N3)	GQ247872	GQ247873
