# Supplementary material for: A Complete Molecular Diagnostic Procedure for Applications in Surveillance and Subtyping of Avian Influenza Virus
Source: Biomed Res Int. 2014 Jun 26;2014:653056. doi: 10.1155/2014/653056 (PMC4098948; doi:10.1155/2014/653056)
Supplement: Supplementary file 1 — Supplemental Table 1 details 25 SYBR-Green qPCR detection primer pairs targeting on 16 HA and 9 NA subtypes of avian influenza. Supplemental Tale 2 lists the detail of 100 primers which are designed for traditional PCR and sequencing purposes for different subtypes of avian influenza virus sequence analysis. [file 653056.f1.pdf]

Supplementary Table 1. List of subtyping primer panel

Primer	Primer Sequence [a] (5' to 3')	Expected product size (bp)
H1qPCR-417f	CATGGCCAAAYCATGAGACAAC	234(417-650)
H1qPCR-650r	ARACRTATGCATCAGYGTTCTGGTA	
H2qPCR-330f	AYCCAGGCAGTTTCAATGATTATG	183(330-512)
H2qPCR-512r	TCTTTGTMARCCARACCATGTTC	
H3qPCR-209f	GRCGRGACTGCACACTRATAGATGC	220(209-428)
H3qPCR-428r	CCATTYTGAGTGACTCCTGTCCA	
H4qPCR-311f	ACTTGYTAYCCATTTGATGTGCCAGA	271(311-581)
H4qPCR-581r	GATGRTGAACTCCCCAKATGTAAAG	
H5qPCR-1475f	CCGCAGTATTCAGAAGARGC	140 (1475-1614)
H5qPCR-1614r	AGACCAGCYAYCATGATTGC	
H6qPCR-696f	GMAGAATTGATTATTAYTGGTCTGT	250(696-945)
H6qPCR-945r	ACATATTTGGGGCATTCTCCWATCCA	
H7qPCR-950f	GGCAACAGGRATGAAGAAYGTTCC	97(950-1046)
H7qPCR-1046r	AATCAGACCTTCCCATCCATTTTC	
H8qPCR-154f	ATTGGAACTGCGAGACTGCA	236(154-389)
H8qPCR-389r	CAGAGCKAGTYACATTCCAYCTGG	
H9qPCR-115f	CACATRCCAAAGAATTRCTCCA	166(115-280)
H9qPCR-280r	CTTTCRACRATGTAGGACCATT	
H10qPCR-1026f	ATAGAAAAYGGATGGGAAGGRAT	300(1026-1325)
H10qPCR-1325r	GTCAATTGTRTGCTGATTYTCCAT	
H11qPCR-259f	ACWTCATGGTCYTACATWGT	243(259-501)
H11qPCR-501r	TGATTGRTGTATCARCCATACCATG	
H12qPCR-165f	CTAGTGCTYGATGACTGTTCATTAGA	209(165-373)
H12qPCR-373r	AAATCAAACATCTTCACTCTTTCATAYT	
H13qPCR-151f	GACWAACCACACAGGAACATATTG	233(151-383)
H13qPCR-383r	TGAAHGATYTGATTCCACTGAACA	
H14qPCR-161f	CACACTGATGAACTGTGCCCAA	193(161-353)
H14qPCR-353r	TGCTTCTGAGACTCTGGTAATCTG	
H15qPCR-184f	TGTGGAATACTGGGAACTATCATTG	237(184-420)
H15qPCR-420r	TGTTCTCTTACACGCACTGGTTG	
H16qPCR-649f	ACAYTRGTATCAACAAAAGARTGGA	242(649-890)
H16qPCR-890r	TGRCAYTTTGTGTTGCATTTRG	
N1qPCR-477f	GTTTGAGTCTGTTGCTTGGTC	190(477-666)
N1qPCR-666r	GCCATTTACACATGCACATTCAG	
N2qPCR-420f	TGCATAGCATGGTCCAGCTCAAG	216(420-635)

N2qPCR-635r	AGCACTTCCATCAGTCATTACTAC	
N3qPCR-614f	ATGACAGACTCCATTAAATCATG	256(614-869)
N3qPCR-869r	TCCAATTGTCCCTACATATRCAGT	
N4qPCR-605f	YATTATGCGAACACAAGAGTC	270(605-874)
N4qPCR-874r	TAATCAAGATCACTGTTGAATC	
N5qPCR-120f	ACAATGAAACTGTRAGGCTGGAA	162(120-281)
N5qPCR-281r	CWATTCTGATYCCATTGTCCTTG	
N6qPCR-232f	TRTGTGAAGTAAACTCATGGCA	181(232-412)
N6qPCR-412r	TCATGTATRGTCCCATTTGCATG	
N7qPCR-442f	GGTCMAGCACHAGTTGCCATGA	172(442-613)
N7qPCR-613r	CCATTGTGRCATACACATTC	
N8qPCR-153f	GGTAACWCAATGGCACAATAC	153(153-305)
N8qPCR-305r	ACATGWCCTCTSGAWCCAATTC	
N9qPCR-521f	AAGYACTAGTTGCCATGATG	206(521-726)
N9qPCR-726r	CAGTGGCRGACCCATCTGTGA	

[a] Codes for mixed bases position: R=A/G, Y=C/T, M=A/C, K=G/T, S=C/G, W=A/T, H=A/C/T.

Supplementary Table 2. Primers list for HA and NA gene segments sequencing of avian influenza A viruses by traditional PCR

Primer	Primer sequence [a] (5' to 3')	Expected product size (bp)	Number of sequences considered for primer design[d]
H1-uni-f	CAAAAGCAGGGGAWAATCAAATCAAYCAA	1124[b] / 1716[c]	74
H1-mid-1123r	CABCCATCTATCATKCCTGTCCATCC		
H1-mid-823f	ATAACRTTYGARGCMACYGGRAA	918	
H1-uni-r	CATTGYAAWGACCCATTAGARCACATCCA		
H2-uni-f	CAAAAGCAGGGGTTATACCATRGACAA	1126 / 1711	67
H2-mid-1118r	TACCARCCATCAAYCATYCCTTGCCA		
H2-mid-842f	GCACCAGAATAYGGWTTCAAAATATC	884	
H2-uni-r	TGCAGAGAYCCRTTRGARCACATCCA		
H3-uni-f	AGCAAAAGCAGGGGATAHTT	1092 / 1736	186
H3-mid-1099r	CCTTCCCATCCATTTTCAATAAA		
H3-mid-932f	ATCACTCCWAATGGDAGCATYCCMAA	813	
H3-uni-r	ACTCAAATGCAAATGTTGCACCTAATRTTGCC		
H4-uni-f	CAAAAGCAGGGGAAACAATGCTATCAAT	907 / 1730	140
H4-mid-884r	GTGTGRCATTTRCTRACRCADGAKCCTAT		
H4-mid-737f	CAAAGYGGYAGRATHAGYTTYTAYTGGAC	999	
H4-uni-r	CAAGGGTGTTTTTTCTYYAAATRCAAATCTG		
H5-uni-f	CAGGGGTTCAATCTGTCAAAATGGA	928 / 1764	1192
H5-mid-911r	GAGTTTATCGCMCCYATTGGAGTTTGA		
H5-mid-815f	CCAGAAWATGCATACAAAATTGTCAARAA	949	
H5-uni-r	ACAAGGGTGTTTTTAACTACAATCTGAACTC		
H6-uni-f	AGCAAAAGCAGGGGAAAATGATTGCA	985 / 1702	261
H6-mid-996r	TTKGGRCATTYYCCWATCCA		
H6-mid-807f	AATGGAAAYCTAATYGCYCCTTGGTATGC	902	
H6-uni-r	TGCATTGAACCATTCGARCACATCCA		
H7-uni-f	CAAAAGCAGGGGATACRAAATGAACA	1082 / 1704	311
H7-mid-1074r	CTTCCCAYCCATTTTCAATRAAWCCHGC		
H7-mid-784f	TTCAATGGGGCHTTYATAGCYCC	920	
H7-uni-r	ACTTATATACAAATAGTGCACCGCATGT		
H8-uni-f	AGCAAAAGCAGGGGTCACAATGGAGAA	947 / 1718	17
H8-mid-920r	GAAAGGGTTTGCTGCTATTRATTGCTCC		
H8-mid-639f	CTRTAYAARAATGCAAACACTTTGAGTAGTGT	1081	
H8-uni-r	AAATACAGAACATGCATCTACAAGATCCAT		
H9-uni-f	AGCAAAAGCAGGGGAA	956 / 1701	318

H9-mid-952r	TTRCTKAYATTKTGRAAHGGCAA		
H9-mid-694f	TTCAAACCADTAATAGGGCCAAGGCC	1008	
H9-uni-r	GCATCTGCAAGATCCATTGGACAT		
H10-uni-f	CAAAAGCAGGGGTCACAATGTACAA	901 / 1695	58
H10-mid-878r	CCYTTCCAAAAACATTTDGATTCACA		
H10-mid-709f	CAAGACCWCARGTSAATGGHCAAAGT	993	
H10-uni-r	AGATTGTGCATCGCATGTTTCCAT		
H11-uni-f	AGCAAAAGCAGGGGAAATATCAAGAAAT	771 / 1731	67
H11-mid-744r	TCCARTARAAKGTCATTCKYCCAGCCT		
H11-mid-569f	TTCAACAACACCAAAGGAAGAGATGT	1162	
H11-uni-r	TTTAAATGCAAATGGTACATCTACATGA		
H12-uni-f	AGCAAAAGCAGGGGTCACAATGGARAARTTCAT	1080 / 1737	25
H12-mid-1049r	CCTTCTATRAARCCWGCAATTGCTCCAAAGAG		
H12-mid-730f	ACARCARGGGAGAATGGATTAYTAYTGGGC	1008	
H12-uni-r	AGTAGAAACAAGGGTGTTTTTAATTAAATACAGA		
H13-uni-f	AGCAAAAGCAGGGGAAAATHTYWAYAA	945 / 1774	25
H13-mid-918r	CCRACMGAWGTCTGRCAYTTRGTRTTGCA		
H13-mid-747f	CCTGGCTATAATGGACAGAGGAGCTGGATGA	1029	
H13-uni-r	AGTAGAAACAAGGGTGTTTTTTTCTAYTTATATACA		
H14-uni-f	GCAGGGGAAAATGATTGCACTCA	974 / 1741	2
H14-mid-945r	TTCCTATAGCAATCCTTGATACATTCTGAAAAGGTTT		
H14-mid-776f	AGTAAACCCAGGGGACTCCA	973	
H14-uni-r	AGTAGAAACAAGGGTGTTTTTTCTTTATATACAGAT		
H15-uni-f	AGGGGAWACAAAATGAACACTCAAATCAT	921 / 1754	5
H15-mid-905r	ACTGTAGAAGCATTCCCCTTCACAT		
H15-mid-780f	ATGGTGCATTCATAGCCCCAGATA	973	
H15-uni-r	AGTAGAAACAAGGGTGTTTTTTCAAATAATTATA		
H16-uni-f	AGCAAAAGCAGGGGATATTGTCAA	1082 / 1762	13
H16-mid-1060r	CAATTGCACCAAACARDCCTCTTTCA		
H16-mid-903f	GGCCAAATGCAACACAAAGTG	865	
H16-uni-r	AGTAGAAACAAGGGTRTTTTTTYCYDAYTATATA		
N1-uni-f	AGCAAAAGCAGGAGWTYAAAATGAATC	929 / 1425	919
N1-mid-1030r	CCAYGGYCKATTYGARSCATGCCA		
N1-mid-675f	CTTGGTCAGCAAGTGCWTGYCATGATG	873	
N1-uni-r	TGTCAATGGTGAATGGCARMTCAGC		
N2-uni-f	AGCAAAAGCAGGAGTGAAAATGAATCCA	699 / 1396	834
N2-mid-714r	TCCTGWGTYCTGAGRATATTTTGRGACCA		
N2-mid-513f	GTTCCATTYCATTTRGGRACCARACAAGT	885	

N2-uni-r	AGGCCATGAGCCTGTTCCA		
N3-uni-f	AGCAAAAGCAGGTGCGAGATGA	926 / 1384	157
N3-mid-978r	GGYCTGTTRGARCCYTTCCARTTGTCYCT		
N3-mid-668f	ATAATRTATGCAGGGAYAATGMSAGACTC	748	
N3-uni-r	ATCCAGGTTCATTGTCTARYCCACAGAA		
N4-uni-f	AGCAAAAGCAGGAGWTTCATAATGAAYCCRAA	1106 / 1442	24
N4-mid-1084r	CTYCCTATCCAAACWCCATCYCCATA		
N4-mid-677f	GAARGGMAAYATYATGCGAACACAAGAGTC	768	
N4-uni-r	TCGAAAAATYACTTGTCTATGTCAAADGGCA		
N5-uni-f	AGCAAAAGCAGGAGTTTAAAATGAATCCA	913 / 1441	47
N5-mid-883r	CTATTCATTCCRTTCCAATTRTCYCKGCAAAC		
N5-mid-724f	TGTTATTGGGTAATGACRGATGGTCC	719	
N5-uni-r	TACATCTTATCGATGTCAAAKGGWAGAATTGC		
N6-uni-f	AGCAAAAGCAGGGTGAAAATGAATCCA	926 / 1421	126
N6-mid-879r	GGTCTATTTGCHCCYTTCCAATTGT		
N6-mid-605f	ACCTAAYAAYAATGCWTCRGCWGTRGTVTGGTA	810	
N6-uni-r	ATGATTTCAGCMCCATCATGCCA		
N7-uni-f	AGCAAAAGCAGGGTGATYGAGAATGAAT	925 / 1412	62
N7-mid-942r	GGYCTRTTWGCYCCYTGCCA		
N7-mid-621f	CARGGRAATAATRAYAAYGCWACAGC	807	
N7-uni-r	ATCGGGGAARGAACCGGA		
N8-uni-f	AGCAAAAGCAGGAGTTTAAAATGAATCCAA	754 / 1411	159
N8-mid-762r	GCKGGTCCRTCHGTCATYACCCA		
N8-mid-597f	TGYCAYGATGGRAAGAARTGGATGA	848	
N8-uni-r	GGAAGAATAGCTCCATCRTGCCA		
N9-uni-f	AGCAAAAGCAGGGTCAAGATGAATCCAAATCA	914 / 1432	63
N9-mid-906r	CCYTGCCAATTRTCYCTRCAYGTGCA		
N9-mid-566f	GGDTGGTCMAGYACWAGYTGYCAYGATGG	880	
N9-uni-r	CTTAGAGRAAGTACTCTATTTTAGCCCCATC		

[a] Codes for mixed bases position: R=A/G, Y=C/T, M=A/C, K=G/T, S=C/G, W=A/T, D=G/A/T, B=G/C/T, H=A/C/T, V=A/C/G.

[b] Product size of partial 1 segment.

[c] Product size of full length segment.

[d] Sequences were retrieved from the GenBank provided by NCBI, USA.
